# The Rise of Imported Dengue Infections in Victoria, Australia, 2010–2016

**DOI:** 10.3390/tropicalmed3010009

**Published:** 2018-01-21

**Authors:** Stacey L. Rowe, Irani Thevarajan, Jack Richards, Katherine Gibney, Cameron P. Simmons

**Affiliations:** 1 Communicable Disease Epidemiology and Surveillance, Department of Health and Human Services, 3000 Melbourne, Australia; Stacey.rowe@dhhs.vic.gov.au (S.L.R.); Katherine.gibney@mh.org.au (K.G.); 2 Victorian Infectious Diseases Service, Royal Melbourne Hospital, Melbourne Health, 3050 Melbourne, Australia; jack.richards@unimelb.edu.au; 3 Peter Doherty Institute, University of Melbourne, 3010 Melbourne, Australia; csimmons@unimelb.edu.au; 4 Disease Elimination Program, Burnet Institute, 3004 Melbourne, Australia

**Keywords:** dengue, travel, epidemiology, healthcare

## Abstract

Dengue notifications have increased dramatically over the past seven years in Victoria, Australia—a trend which has been seen nationally and reflects increased cases internationally. We reviewed the epidemiology of dengue among Victorian travellers, changes in diagnostic methods and describe the burden placed on local health systems resulting from this disease of public health importance. Cases of dengue notified to the Department of Health and Human Services in Victoria, Australia, between 1 January 2010 and 31 December 2016 were included in this review. Demographic, clinical, diagnostic methods, and risk factor data were examined using descriptive epidemiological analyses. Cases of dengue increased on average by 22% per year, with a total of 2187 cases (5.5 cases/100,000 population) notified over the 7-year reporting period. The most frequently reported country of acquisition was Indonesia (45%) followed by Thailand (14%). The use of multiple diagnostic methods, including the non-structural protein 1 antigen (NS1Ag) detection test, increased over time. The median time between onset of illness and diagnosis diminished from 9 days (IQR: 2–15) in 2010 to 4 days (IQR: 2–7) in 2016. Proportionally more cases were discharged directly from emergency departments in recent years (10% in 2010 to 28% in 2016, *p* < 0.001).The increasing incidence of dengue in Australia is reflective of its growing prominence as a travel medicine problem in western countries. For travellers with non-severe dengue, the improved timeliness of dengue diagnostics allows for consideration of best practice ambulatory management approaches as used in endemic areas.

## 1. Introduction

Dengue is endemic in more than 100 countries worldwide, with the Americas, South-East Asia and Western Pacific regions most affected. Infection with dengue is a leading cause of morbidity in endemic countries, and presents a significant cause of illness in travellers returning from dengue-endemic regions. There is ongoing global expansion of this disease with approximately 390 million infections annually, of which 100 million are symptomatic [1]. At present, there is no specific chemoprophylaxis or therapy for dengue infection, but a dengue vaccine is available. 

Dengue infection is caused by an RNA virus that belongs to the genus *Flavivirus* and there are four dengue virus serotypes. Transmission occurs through the bite of an infected mosquito, with *Aedes* (*Ae*) *aegypti* primary vector of dengue viruses, but *Ae. albopictus* is an important secondary vector. Most primary infections are asymptomatic [2]. Clinical disease usually presents as an acute febrile illness with accompanying myalgia, headache and rash [3]. Disease is usually self-limiting, however severe disease occurs in about 2–3% of symptomatic paediatric cases in endemic settings; a lower proportion of cases result in severe disease among adults [4,5,6]. Severe dengue occurs infrequently in international travellers, and is estimated to comprise in 1–3% of dengue infections in this group [3,7,8].

Travellers are known to play a key role in the introduction of dengue virus infections to dengue-naïve destinations where suitable local vectors are present for autochthonous transmission. In particular, travellers who have acquired infection in an endemic setting may cross international borders whilst in the pre-symptomatic phase and serve as index cases for new outbreaks, particularly in locations where there is minimal population immunity [9].

Dengue infection in returned travellers has been increasingly reported from all regions of the world [10]. This trend has also been observed in Australia in recent years with a marked increase in notified cases of dengue infection from 2010 onwards [11]. Whilst locally-acquired infection occurs in Northern Queensland, a majority of infections have occurred in returned travellers from Asian destinations, with most of the overseas-acquired cases occurring in Indonesia [11].

National surveillance of dengue in Australia was established in 1991. In Victoria, the Public Health and Wellbeing Act 2008 (and its associated Public Health and Wellbeing Regulations 2009) requires that medical practitioners and laboratories notify the Department of Health and Human Services of a person who has or may have dengue. Notified cases are classified according to the Communicable Disease Network Australia national surveillance case definition for dengue applicable at the time of notification (http://www.health.gov.au/internet/main/publishing.nsf/content/cda-surveil-nndss-casedefs-cd_dengue.htm) (Box 1). The diagnostic tests that are available to diagnose dengue cases include serological assays, molecular diagnostics and antigen detection assays. There are differences between the diagnostic yield depending on the assays used and the timing of the test. In the first week of illness, dengue virus RNA or non-structural protein 1 antigen (NS1Ag) can be detected by PCR or antigen detection tests respectively, while IgM and IgG antibodies are more likely to be detected a week or more after illness onset.

This review characterizes the epidemiology of imported dengue infections among Victorian travellers. The changing trends in dengue infections over time highlights the growing burden placed on local health systems.

## 2. Methods

Confirmed and probable case definitions are described in Box 1. Cases of dengue notified to the Department of Health and Human Services, Victoria, Australia, between 1 January 2010 and 31 December 2016 were extracted from the Public Health Events Surveillance System (PHESS). Variables extracted included: notification date, case classification (probable or confirmed), age, sex, country of birth, details of notified laboratory tests including specimen collection date, test types and test results with dengue serotype when reported, date of symptom onset, symptoms, and type of medical care sought. Place of dengue acquisition was determined from the travel history during the 4–14 days prior to illness onset. If multiple countries with dengue transmission were visited during this period, the international destination in which the case spent the most days during the 4–14 days prior to illness onset was assumed to be the country of acquisition. 

We examined duration of symptoms before diagnosis, as well as the time between seeking care and notification to the department. The date of specimen collection was used as a proxy for the date of diagnosis. Duration of symptoms before diagnosis was calculated by subtracting specimen collection date from the date of symptom onset (as reported by the case or their notifying medical practitioner). Time to notification was calculated by subtracting notification date from the specimen collection date. 

Statistics were presented as frequencies with percentages, and medians with interquartile range (IQR) where relevant. Crude notification incidence rates were calculated using the Victorian Estimated Resident Population (ERP) for each year, and adjusted incidence rates were calculated by direct standardisation using 2015 ERP as the reference population. Unless otherwise specified, the data were summarised as aggregates of the seven-year period from 2010–2016. Poisson regression was used to describe average annual change in notification numbers, and Chi square test was used to describe differences between proportions. All analyses were carried out in Stata Statistical Software: Release 14.

Box 1 **Dengue Virus infection—Australian National Notifiable Disease Surveillance System Case Definition.**

**Effective date:** 1 January 2013–31 December 2016

Confirmed and probable cases should be notified

**Confirmed case:** Requires laboratory definitive evidence AND clinical evidence

**Laboratory definitive evidence:** Isolation of dengue virus OR detection of dengue virus by nucleic acid testing OR detection of non-structural protein 1 (NS1) antigen in blood by EIA OR IgG seroconversion or a significant increase in antibody level or a fourfold or greater rise in titre to dengue virus, proven by neutralisation or another specific test OR detection of dengue virus-specific IgM in cerebrospinal fluid, in the absence of IgM to Murray Valley encephalitis, West Nile virus /Kunjin, or Japanese encephalitis viruses. **Clinical evidence:** A clinically compatible illness (e.g., fever, headache, arthralgia, myalgia, rash, nausea/vomiting, with a possible progression to severe plasma leakage, severe haemorrhage, or severe organ impairment CNS, liver, heart or other).

 

**Probable case:** Requires laboratory suggestive evidence AND clinical evidence AND epidemiological evidence

**Laboratory suggestive evidence:** Detection of dengue virus-specific IgM in blood

**Clinical evidence:** As for confirmed case

**Epidemiological evidence:** A plausible explanation, e.g., travel to a country with known dengue activity OR exposure in Australia where local transmission has been documented within the previous month.

 

**Effective date:** 1 January 2010–31 December 2012

Only confirmed cases should be notified

**Confirmed case:** Requires laboratory definitive evidence AND clinical evidence

**Laboratory definitive evidence:** Isolation of dengue virus OR detection of dengue virus by nucleic acid testing OR IgG seroconversion or a significant increase in antibody level or a fourfold or greater rise in titre to dengue virus, proven by neutralisation or another specific test OR detection of dengue virus-specific IgM in cerebrospinal fluid, in the absence of IgM to Murray Valley encephalitis, West Nile virus/Kunjin, or Japanese encephalitis viruses OR detection of dengue virus-specific IgM in serum, except in North Queensland. In North Queensland, dengue-specific IgM in serum is acceptable evidence ONLY when this occurs during a proven outbreak.

**Clinical evidence:** A clinically compatible illness (e.g., fever, headache, arthralgia, myalgia, rash, nausea/vomiting, with a possible progression to dengue haemorrhagic fever, dengue shock syndrome or meningoencephalitis).

## 3. Results

### 3.1. Increase in Dengue Notifications

**Figure 1:**
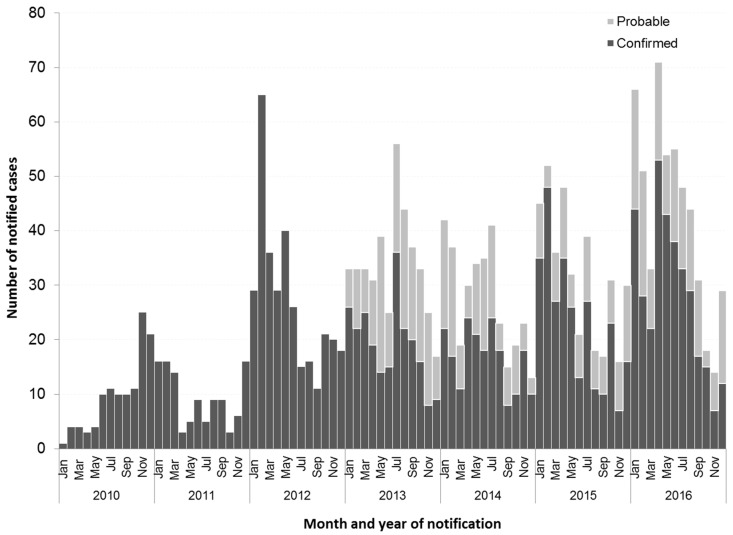
Number of notifications, confirmed and probable dengue cases 2010–2016, Victoria.

Between 1 January 2010 and 31 December 2016, 2187 cases of dengue were notified to the Victorian Department of Health and Human Services, 73% of which were classified as confirmed. Notified dengue cases increased more than four-fold between 2010 (114 cases) and 2016 (516 cases). The median number of cases notified per month was 25 (IQR: 14–26) and ranged from 1 to 71 (Figure 1). Higher proportions of cases were notified in the summer and autumn months, although a late peak was observed in July 2013.

The overall notification incidence was 5.5 cases per 100,000 population, and most cases (1827, 84%) were residents of metropolitan Victoria (which includes Melbourne city and surrounding metropolitan suburbs. (https://liveinmelbourne.vic.gov.au/discover/melbourne-victoria/metropolitan-melbourne).

**Figure 2:**
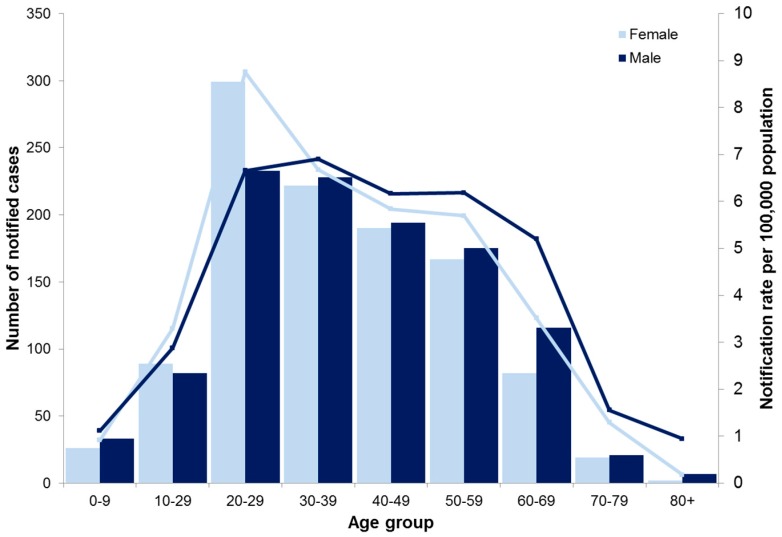
Number and notification rate of dengue cases, by age and sex, 2010–2016, Victoria.

The median age was 36 years (IQR: 26–51 years) and the sex distribution was even. The highest notification incidence was observed in females aged 20–29 years (8.8 cases/100,000) (Figure 2). Overall, 41% of cases were born in Australia and 16% were overseas-born; data relating to country of birth was missing in the remaining 42% of cases. Over time, the proportion of cases being notified in overseas-born Victorian residents has increased significantly from 10.8% of cases in 2011 to 20.2% of cases in 2016 (*p* < 0.001).

### 3.2. Country of Acquisition

**Figure 3:**
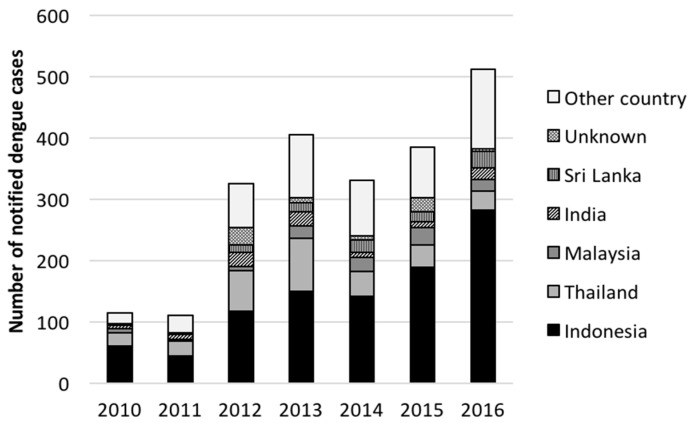
Number of notified cases by year and country of acquisition, Victoria 2010–2016.

More than half the notified dengue cases were acquired in either Indonesia (985 cases (45%)) or Thailand (305, (14%)). Malaysia (108 (5%)), India (97 (4%)) and Sri Lanka (91 (4%)) were the next most frequent countries of acquisition (Figure 3a). Information on place of acquisition was missing for 74 cases (3%). The remaining 525 cases (24%) were acquired in other countries, including three cases in 2011 that acquired their illness in northern Queensland, Australia. Ninety-six (4%) cases reported more than one potential country of acquisition.

The number of notified dengue cases acquired in Indonesia ranged from 44 in 2011 to 282 in 2016 (Figure 3a), while the proportion acquired in Indonesia ranged from 36% in 2012 to 55% in 2016. The greatest number of dengue cases acquired in Thailand were notified in 2013 (87 cases), with the proportion acquired in Thailand ranging from 6% (2016) to 22% (2011). Cases acquired in Indonesia increased on-average 30% per year while there was not a consistent change in cases acquired in Thailand (Table 1)

### 3.3. Diagnostic Tests

**Figure 4:**
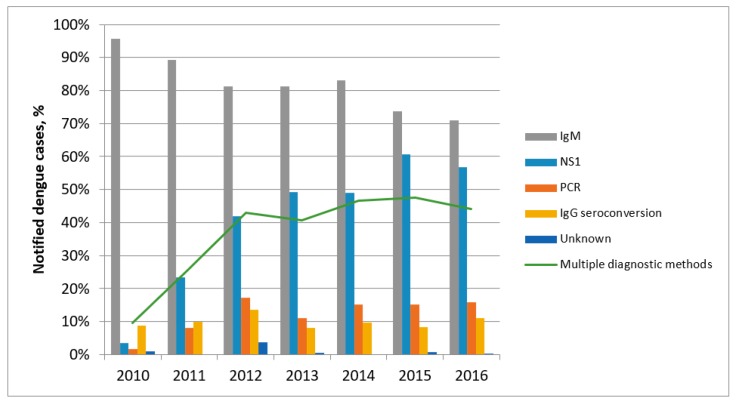
Proportion of notified dengue cases by diagnostic method and year, Victoria 2010–2016.

In 2010, 96% of notified dengue cases had IgM detection documented, while NS1Ag detection was documented in only 4% and a positive PCR test in only 2% of notified cases. Both NS1Ag detection and PCR testing became more common after 2011 (Figure 4); by 2016, NS1Ag detection was documented for 57% of notified cases, positive PCR test in 16%, while documented IgM detection had fallen to 71% of notified cases (Figure 4). Overall, 10% of cases had documented IgG seroconversion, and this remained stable over the study period. The proportion of cases diagnosed using multiple methods increased from 10% in 2010 to 44% in 2016. Method of diagnosis was unable to be determined for 20 (0.9%) cases. The infecting serotype was determined in 552 (25.3%) cases. Serogroup 2 (207 cases (37.5% of cases with serogroup identified)) was more common than serogroup 1 (171 cases (31.0%)) and serogroup 3 (138 cases (25.0%)), while serogroup 4 was uncommon. From 2010–2015, only 15 serogroup 4 cases were notified (annual range 0–5 cases), while 21 serogroup 4 cases were notified in 2016. 

### 3.4. Clinical Presentation

Data relating to symptoms were available in 1642 (75%) cases. More than two-thirds of cases reported fever (69.6%), and more than half of the cases reported myalgia (52.8%) and headache (51.4%). Other commonly-reported symptoms included arthralgia (43.6%), rash (38.4%), loss of appetite (34.5%) and chills/rigors (31.0%). Two cases were reported with septic shock, one of whom required admission to an intensive care unit. No cases were reported as having died due to their infection; a 91-year-old female was reported to have died due to other, unknown causes. 

**Figure 5:**
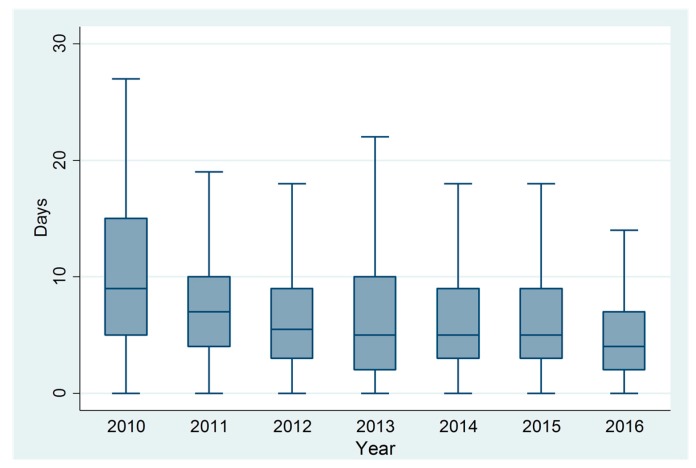
Days (median, IQR) between onset of illness and seeking medical care, 2010 to 2016.

Duration of symptoms before diagnosis was calculated in 1673 cases (76.5%). Overall, the median time between onset of symptoms and diagnosis was 5 days (IQR: 3–9). Over time, this declined from 9 days (IQR: 2–15) in 2010 to 4 days (IQR: 2–7) in 2016 (Figure 5). The interval was shorter for cases diagnosed by NS1Ag detection and PCR (4 days (IQR 4–6 days)) and longer for those diagnosed by serology (IgM detection: 7 days (IQR 4–12 days), IgG detection: 5 days (IQR 5–19 days)). The median number of days from specimen collection date to being notified to the department was 7 days (IQR: 5–9), and this did not demonstrably vary over time.

**Figure 6:**
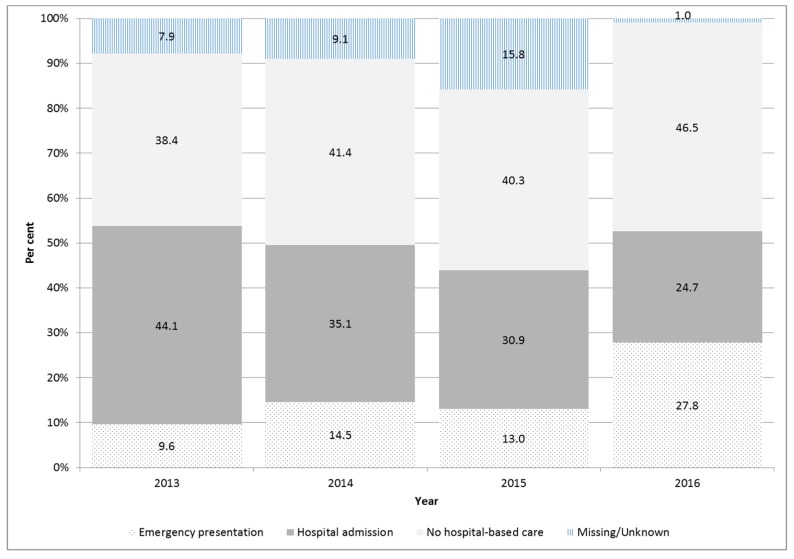
Proportion and type of medical care sought by dengue cases, Victoria 2013–2016.

The type of medical care that cases sought was reliably captured for 1636 cases notified between 2013 and 2016. Overall, half of the cases reported seeking hospital-based care, including 541 (33%) cases reporting being admitted to hospital and 280 (17%) reporting presentation to an emergency department only (Figure 6). Forty-two per cent of cases reported that they did not seek hospital-based care for their infection, and for the remaining 128 (7%) cases, the type of medical care a case sought was missing or unknown. The proportion of cases being admitted to hospital significantly declined over time: from 44% in 2013 to 25% in 2016. Conversely, the proportion of cases who presented only to an emergency department significantly increased: from 10% in 2013 to 28% in 2016 (*p* < 0.001). The proportion of cases who sought non hospital-based care was relatively stable over time, averaging 42% over the four years (Figure 6). 

Of the cases seeking hospital-based care, 772 (94%) did so at a metropolitan hospital, including 65 cases who were residents of rural Victoria. Of the cases admitted to hospital, length of stay was able to be calculated for 467 (86%) cases. Of those, the total number of bed days was 1654, and the median length of stay was 3 days (IQR 2–4).

## 4. Discussion

Cases of dengue infection have risen dramatically in recent years—a trend that has been observed locally, nationally [11] and globally [1].We reviewed surveillance data collected by the Victorian Department of Health and Human Services to describe the changing epidemiology of dengue among Victorian residents between 2010 and 2016. Our review identified an on-average 22% increase in the number of dengue cases being notified each year, with more than 500 cases notified in 2016—the highest number of cases notified in Victoria since the onset of disease notification. In recent years, cases were diagnosed earlier in their illness and a higher proportion was discharged directly from the emergency department into the community.

With the exception of three cases that acquired their illness in northern Queensland, Australia, all cases notified were acquired following overseas travel. The most commonly reported country of travel among cases in this review was Indonesia followed by Thailand. This finding is consistent with national data, which showed that 66% of cases reportedly acquiring their infection in Indonesia [11]. Travel to Indonesia by Australian travellers has shown the strongest growth in the last decade and is the second most common tourist destination [12]. The changing epidemiology of dengue infection in Victorian residents is likely to be multifactorial. First, it is likely to be reflective of the substantial dengue disease burden in regions of South-East Asia and the Western Pacific, coupled with an increasing propensity for Australians to travel to these destinations. Second, the seasonal fluctuations observed in our data may reflect the preference for Australians to travel during warmer weather in the Southern Hemisphere, which also coincides with Christmas and other cultural celebrations in the region [12]. Third, these temporal trends may also be driven by variations in transmission risk in Indonesia, which is heightened during the rainy season between October and March. Consistent with our findings, a study of dengue infections in Australian travellers identified that 70% report a history of travel to Asian destinations [2].Risks posed by dengue should therefore be considered by travellers before and during travel, and medical practitioners and healthcare systems need to accommodate for the changes in disease occurrence in travellers in view of these factors.

With the exception of three cases that acquired their illness in northern Queensland, Australia, all cases notified were acquired following overseas travel. The most commonly reported country of travel among cases in this review was Indonesia followed by Thailand. This finding is consistent with national data, which showed that 66% of cases reportedly acquiring their infection in Indonesia [11]. Travel to Indonesia by Australian travellers has shown the strongest growth in the last decade and is the second most common tourist destination [12]. The changing epidemiology of dengue infection in Victorian residents is likely to be multifactorial. First, it is likely to be reflective of the substantial dengue disease burden in regions of South-East Asia and the Western Pacific, coupled with an increasing propensity for Australians to travel to these destinations. Second, the seasonal fluctuations observed in our data may reflect the preference for Australians to travel during warmer weather in the Southern Hemisphere, which also coincides with Christmas and other cultural celebrations in the region [12]. Third, these temporal trends may also be driven by variations in transmission risk in Indonesia, which is heightened during the rainy season between October and March. Consistent with our findings, a study of dengue infections in Australian travellers identified that 70% report a history of travel to Asian destinations [2].Risks posed by dengue should therefore be considered by travellers before and during travel, and medical practitioners and healthcare systems need to accommodate for the changes in disease occurrence in travellers in view of these factors.

Changes in testing practices may also have influenced the increase in dengue notifications among Victorian residents. The increasing availability and use of diagnostic tests—such as the NS1 antigen detection—in addition to the increasing use of more than one type of test used for diagnoses may have contributed to the observed increase in notified cases through more complete case ascertainment. In order to assess the influence of changes in testing practices on dengue notifications, denominator data relating to the total number of tests ordered for dengue in Victoria would be required in addition to the numerator data obtained through notifications made to the Department of Health and Human Services under the existing passive surveillance system. However, we were unable to quantify this as denominator data were not available in this study. Improved awareness amongst medical practitioners of dengue as a possible diagnosis in Victorian travellers returning from overseas may also have improved case ascertainment and influenced the increase in dengue notifications observed in this study. During the study period (February 2013), the Victorian Chief Health Officer issued an alert for health professionals to advise of an increase in dengue being notified from travellers returning from overseas, and to remind them to consider this diagnosis in returned travellers with clinically-compatible symptoms [13]. This may have led to increased awareness and testing of travellers amongst medical practitioners.

Approximately half of the cases in our study presented to Victorian hospitals, and subsequent admission was common. Interestingly, there was an increase in the proportion of cases that are being discharged directly from the emergency department. As demonstrated in this review, the diagnostics for dengue infections have changed over the study period and direct discharge from the emergency department maybe reflective of improved availability and timeliness of diagnostic techniques such as NS1 antigen detection and other rapid diagnostics tests. The earlier diagnosis leading to earlier discharge from hospital raises the possibility that travellers are being discharged in the critical phase of their illness (days three to seven and typically coincides with defervescence) highlighting the importance of identification of patients that are suitable for safe outpatient care.

Cases presenting for hospital-based care largely did so at metropolitan hospitals with 84% of dengue notifications originating from residents of metropolitan Victoria. For cases that were admitted to hospital, the median length of stay was three days. This has important implications for health resource allocation and economic burden, particularly if the trend of increased notifications continues. Febrile returned travellers are often admitted to hospital to initiate a diagnostic work-up inclusive of tests for respiratory, gastrointestinal and vector-borne infections, and to institute the appropriate management. The availability of rapid diagnostic tests for dengue, that typically have a turn-around time under five hours, could expedite the diagnostic work-up in the febrile returned traveller. If confirmed to be mild dengue infection, carefully selected patients who do not have warning signs, have no or minimal co-morbidities and are not co-infected with other travel-related infections, could be managed as an outpatient with daily review during the critical phase of illness. It would be important to implement clinical guidelines for the inpatient and outpatient management including the recognition of warning signs that may predict the development of severe dengue. In a recent review of hospitalised dengue-infected patients within Australia, 40% were classified as dengue infection with warning signs and only one patient developed severe dengue [14]. In endemic countries, dengue is essentially managed in the outpatient setting both as a result of the relatively non-severe clinical spectrum of the infection and also due to the limitations in hospital-based resources.

People are most infective during the first three days of viraemia, and if bitten by suitable vectors during that time, are able to transmit to mosquitoes. In Victoria, there is no established mosquito vector to enable dengue transmission locally; however, the importation of *A. aegypti* into Victoria’s Melbourne international airport in 2014 and the detection at Melbourne international airport of *Ae. albopictus* in 2012, raise concerns about the theoretical risk of local transmission. Whilst *Ae. aegypti* is typically intolerant of cooler climates, *Ae. Albopictus* can tolerate cooler climatic conditions, and is thought to be responsible for autochthonous cases in Japan [15] and France [16]. *Ae. albopicus* is not currently present on mainland Australia; however, its detection in Melbourne, highlights the importance of surveillance and quarantine practices at international ports of entry [17].

There is one licensed vaccine for dengue, a live attenuated tetravalent vaccine developed and marketed by Sanofi Pasteur, but it has a complex efficacy profile with suboptimal efficacy against serotype 1 and 2 and poor efficacy in flavivirus-naïve children, and is not currently recommended for travellers (http://www.who.int/immunization/policy/sage/sage_wg_dengue_mar2015/en/). Other live vaccines are in phase III trials, results of which will become available in 2019/2020 [18,19]. In the absence of a vaccine, the mainstay of prevention for dengue is personal protective practices such as use of mosquito repellents and protective clothing particularly during the peak *Ae. aegypti* biting periods of early morning and dusk.

The increasing trend in the number of dengue cases being notified among Victorians returning from international travel has implications at an individual level, for the medical community, and for public health authorities. Improved awareness of the risk of travel-related dengue in popular tourist destinations is required to encourage individuals to adopt personal protective strategies against mosquito bites when traveling, such as the use of suitable mosquito repellents and wearing long, loose-fitting clothing. The increasing burden placed on health services requires careful consideration of the optimal allocation of healthcare resources for the investigation and management of this infection. A transition from an inpatient to an outpatient community-based care model for dengue-infected travellers would reduce the current and projected burden on the healthcare system. The availability of rapid diagnostics coupled with a management approach that selects travellers without features that are associated with the development of severe dengue enables the consideration of an outpatient community-based care. Inherent to this model is optimal patient education, including warning signs that need to be self-monitored and the ability to attend follow-up assessments. There is a wealth of experience in the management of non-severe dengue in the community in endemic countries that can be instructive for this setting, where better resource allocation can be considered.

## Figures and Tables

**Table 1:** Change in dengue notification incidence per year, Victoria 2010–2016.

Country of Acquisition	IRR *	[95% Confidence Interval]
All	1.22	[1.19–1.25]
Indonesia	1.30	[1.26–1.34]
Thailand	1.02	[0.97–1.08]
Other (excl. Indonesia or Thailand)	1.25	[1.21–1.30]

* IRR—incidence rate ratio.
